# Construction of an Emotional Lexicon of Patients With Breast Cancer: Development and Sentiment Analysis

**DOI:** 10.2196/44897

**Published:** 2023-09-12

**Authors:** Chaixiu Li, Jiaqi Fu, Jie Lai, Lijun Sun, Chunlan Zhou, Wenji Li, Biao Jian, Shisi Deng, Yujie Zhang, Zihan Guo, Yusheng Liu, Yanni Zhou, Shihui Xie, Mingyue Hou, Ru Wang, Qinjie Chen, Yanni Wu

**Affiliations:** 1 Nanfang Hospital Southern Medical University Guangzhou China; 2 School of Nursing Southern Medical University Guangzhou China; 3 China Electronic Product Reliability and Environmental Testing Institute Guangzhou China

**Keywords:** breast cancer, lexicon construction, domain emotional lexicon, sentiment analysis, natural language processing

## Abstract

**Background:**

The innovative method of sentiment analysis based on an emotional lexicon shows prominent advantages in capturing emotional information, such as individual attitudes, experiences, and needs, which provides a new perspective and method for emotion recognition and management for patients with breast cancer (BC). However, at present, sentiment analysis in the field of BC is limited, and there is no emotional lexicon for this field. Therefore, it is necessary to construct an emotional lexicon that conforms to the characteristics of patients with BC so as to provide a new tool for accurate identification and analysis of the patients’ emotions and a new method for their personalized emotion management.

**Objective:**

This study aimed to construct an emotional lexicon of patients with BC.

**Methods:**

Emotional words were obtained by merging the words in 2 general sentiment lexicons, the Chinese Linguistic Inquiry and Word Count (C-LIWC) and HowNet, and the words in text corpora acquired from patients with BC via Weibo, semistructured interviews, and expressive writing. The lexicon was constructed using manual annotation and classification under the guidance of Russell’s valence-arousal space. Ekman’s basic emotional categories, Lazarus’ cognitive appraisal theory of emotion, and a qualitative text analysis based on the text corpora of patients with BC were combined to determine the fine-grained emotional categories of the lexicon we constructed. Precision, recall, and the F1-score were used to evaluate the lexicon’s performance.

**Results:**

The text corpora collected from patients in different stages of BC included 150 written materials, 17 interviews, and 6689 original posts and comments from Weibo, with a total of 1,923,593 Chinese characters. The emotional lexicon of patients with BC contained 9357 words and covered 8 fine-grained emotional categories: joy, anger, sadness, fear, disgust, surprise, somatic symptoms, and BC terminology. Experimental results showed that precision, recall, and the F1-score of positive emotional words were 98.42%, 99.73%, and 99.07%, respectively, and those of negative emotional words were 99.73%, 98.38%, and 99.05%, respectively, which all significantly outperformed the C-LIWC and HowNet.

**Conclusions:**

The emotional lexicon with fine-grained emotional categories conforms to the characteristics of patients with BC. Its performance related to identifying and classifying domain-specific emotional words in BC is better compared to the C-LIWC and HowNet. This lexicon not only provides a new tool for sentiment analysis in the field of BC but also provides a new perspective for recognizing the specific emotional state and needs of patients with BC and formulating tailored emotional management plans.

## Introduction

### Background

In 2020, breast cancer (BC) became the most commonly diagnosed cancer in the world, and there were more than 2.26 million new cases of BC and almost 685,000 deaths from BC worldwide [1]. The diagnosis of BC usually occurs at a stage when women are in the middle of career development or child-rearing and unprepared or unable to cope with the risk of lifelong recurrence and death [2]. Notably, the treatment side effects, together with prognostic uncertainty, cause patients to suffer negative emotional experiences, such as body image disturbance [3] and recurrence and heredity confusion [4], which have been negatively associated with treatment adherence and the quality of life [2]. There has been increasing attention placed on early identification and treatment of emotional distress in patients with cancer, which has been regarded as the “sixth vital sign” [5].

In Chinese culture, free expression of emotions, especially negative ones, may temporarily disrupt group harmony [6,7]. Chinese women, especially, are conflicted about disclosing emotional distress and experience high levels of ambivalence about doing so [8,9]. Meanwhile, socially constrained responses may be negatively associated with relationship satisfaction, aggravate self-stigmatization, and result in persistent emotional distress and reduced self-efficacy in coping with stress [7]. These conditions are not conducive to health care professionals’ and caregivers’ timely identification of patients’ emotions or their ability to provide corresponding emotional support. Furthermore, owing to the lack of mental health knowledge, assessment tools with interference, and patient resistance because of cancer-related stigma [10,11], there are some insurmountable obstacles in the emotional management and psychological care of patients with BC.

Extensive research in psychology has shown that word use and linguistic features can reflect individuals’ thoughts, emotions, and experiences and thus can be used to identify their social and psychological states [12,13]. For example, numerous studies have shown that expressive writing (EW) and interviews are effective ways to listen to the patients’ voice [10,14]. Furthermore, recently, social media platforms, such as Facebook, Twitter, and Weibo, have emerged as a rich yet largely untapped resource for understanding what patients are frankly saying about their experiences and thoughts, which has provided a new breakthrough point for people’s sentiment analysis [15-17]. Therefore, for people who have little chance or who do not take the initiative to disclose their mental conditions to health care professionals, we can capture their emotional expressions from their written, verbal, or online text materials. Thus, interventions and more targeted treatments can be administered to patients with potential emotional distress.

Sentiment analysis is the computational study of an individual’s opinions, emotions, and attitudes [18], which is an effective method that helps analyze and interpret enormous amounts of data and information, thereby identifying and extracting people’s opinions and emotions [16,19,20]. Research on individual emotions, spirit, and psychological detection based on deep learning and emotional lexicons is increasing [21-24]. The lexicon-based method of sentiment analysis may be the simplest and most basic method to analyze emotional polarity [18,19,25]. An emotional lexicon, which consists of a list of sentiment words or phrases, as well as their sentiment polarities and intensities, is the most important component in sentiment analysis systems and plays an important role in sentiment analysis tasks with different text granularities, such as words, phrases, and sentences [26,27]. Although the efficiency of sentiment analysis based on machine learning is high, the model training in this method is highly dependent on the quantity and quality of labeled data sets [20,28,29]. Almost all these data sets come from the internet, ignoring the emotional text information generated in other forms by individuals [30,31]. Therefore, in the absence of a sufficient high-quality training corpus, the lexicon-based method of sentiment analysis has more advantages.

The innovative method of sentiment analysis based on an emotional lexicon has prominent advantages in capturing emotional information, such as individual attitudes, experiences, and needs, which provides a new perspective and method for emotion recognition and management for patients with BC. However, at present, sentiment analysis in the field of BC is limited, and there is no emotional lexicon for this field. Therefore, it is necessary to construct an emotional lexicon that conforms to the characteristics of patients with BC so as to fill the missing gaps in sentiment analysis of BC and provide a new tool for accurate identification and analysis of patients’ emotions and a new method for their personalized emotion management. This study aimed to manually construct an emotional lexicon for patients with BC, which can be uploaded into mainstream text analytic software (eg, Linguistic Inquiry and Word Count [LIWC]-22) to help researchers identify and analyze terms associated with emotions in a text-based corpus or writing content (eg, news, diary) so as to understand the expressions of those emotions embedded in spoken and written languages.

### Related Work

#### Sentiment Analysis Approaches

Many sentiment analysis approaches have been proposed in the past few years. Polarity detection is the most common form of sentiment analysis, which can be classified into 3 main types: lexicon-based approaches, supervised learning methods, or semisupervised learning methods [18,19,25]. Numerous state-of-the-art approaches to sentiment analysis rely on supervised or semisupervised learning techniques [18], but both approaches may be combined into hybrid methods as well. Although sentiment analysis based on supervised or semisupervised learning methods and a general emotional lexicon is common, some studies have pointed out that they cannot effectively analyze texts in specific fields, such as health care [32-34]. These tools, albeit extremely cost-efficient and versatile in certain analytic settings, have problems, such as a lack of sufficient details regarding algorithm development, limited use for task- and theory-specific research, and the requirement of highly structured data sets [32,35]. Moreover, sufficient labeled training data are required in supervised learning methods for sentiment analysis, and training data acquisition becomes a laborious process [33,36].

Lexicon-based sentiment analysis methods tend to sacrifice computational efficiency for classification accuracy, which is typically inferior to the classification accuracy of machine learning techniques in specific domains in which machine learning models can be trained and optimized [32,34]. Surprisingly, lexicon-based methods have an attractive advantage over machine learning methods in that they have more robust performance across domains and texts and can be generalized relatively easily to other languages using lexicons [34]. Additionally, lexicon-based methods enable deep linguistic analysis to be incorporated into the sentiment analysis process [25], which, if fine-tuned, can improve classification accuracy.

#### Sentiment Analysis in the Field of BC

The traditional clinical diagnosis of patients’ psychological problems requires not only filling in some evaluation scales, such as the Self-Rating Depression Scale, but also conducting 1-on-1 interviews and long-term observation [5,11,20], which may have limitations in detection efficiency, since all require good cooperation from patients [20]. Fortunately, things are changing with the development of sentiment analysis. For example, Cabling et al [31] conducted sentiment analysis of an online BC support group regarding tamoxifen to understand users’ emotions and opinions. Clark et al [37] investigated over 48,000 BC-related tweets using econometric sentiment analysis to quantitatively extract emotionally charged topics. Praveen et al [38] used advanced machine learning techniques to understand and analyze the attitudes of people who have survived BC, while discussing their experience of surviving and the stress associated with it. Compared with traditional approaches, these methods have been proven effective and inexpensive and have been shown to reduce limitations and assist in clinical diagnosis in a more flexible way.

#### Research on Emotional Lexicon Construction

An emotional lexicon is a collection of words or phrases that convey feelings [18,20]. It contains words and assigns sentiment scores or classes to single terms. Each entry in an emotional lexicon is associated with its sentiment orientation or strength [18,26]. Entries in a lexicon can be divided into categories according to their sentiment orientations, such as positive or negative [26,39]. There are several well-known general emotional lexicons, such as the Chinese Linguistic Inquiry and Word Count (C-LIWC) [40], HowNet [41], and the General Inquirer (GI) [42]. Emotional lexicons are constructed manually or automatically [20,27,36,43,44].

In the manual method, expert annotators annotate the emotional polarity and classification of words [36]. Some widely used emotional lexicons have been constructed for emotion analysis and application manually or semiautomatically [36,39,42,45]. For example, the GI lexicon [42] provides a binary classification (positive/negative) of approximately 4000 sentiment-bearing words manually annotated. The Affective Norms for English Words (ANEW) [45] provides valence scores for roughly 1000 words manually assigned by several annotators. Similarly, the Semantic Orientation CALculator (SO-CAL) entries [26] consist of roughly 4000 words manually tagged by a small number of linguists with a multiclass label (from very negative to very positive). In addition, the Dictionary of Affect in Language (DAL) contains roughly 9000 words manually rated along the dimensions of pleasantness, activation, and imagery [46].

The automatic method involves calculating the polarity and intensity of emotional words using some algorithms, such as co-occurrence information and context information in the corpus, to construct an emotional lexicon [20,26,27,43]. For example, Yang et al [47] constructed a hotel sentiment lexicon based on users’ behavior and the improved Semantic Orientation Pointwise Mutual Information (SO-PMI) algorithm and then used the lexicon for feature extraction contrast. Li et al [43] proposed a deep learning–based framework to construct a Chinese financial domain sentiment lexicon, using word vector models and deep learning–based classifiers in the process. Additionally, Li et al [20] extracted sentiment words from WordNet-Affect and calculated the co-occurrence frequency between the words and each emoji constructed in their manually labeled emoji sentiment lexicon in order to automatically expand the lexicon. Furthermore, Chao et al [27] proposed a semisupervised sentiment orientation classification algorithm based on Word2Vec and obtained a lexicon in different areas efficiently.

Both lexicons constructed with these 2 methods (manual or automatic) have shown satisfactory results. Although the time and cost associated with annotation tasks are high, the highest precision is obtained with manual annotation and it is deemed more accurate than other methods [36,44]. Automatically created training corpora are usually larger, but the precision is highly dependent on the machine learning algorithm [32,34,36]. Furthermore, the manual method can fully consider the polysemy property of words and the indistinctness property of sentiment categories according to the context of words [44,48]. Conversely, in some cases, the automatic method fails to extract some implicit features or aspects of the special-domain text [26,35,36]. Therefore, considering the particularity of emotional expression in the text corpus of BC mentioned before, we finally decided to manually construct a BC domain–specific emotional lexicon.

#### General Emotional Lexicons: C-LIWC and HowNet

A text analysis application LIWC has been developed to provide a better method for studying verbal and written text samples [40,49,50]. The LIWC, including a software program and a lexicon, is one of the most well-known lexicons in quantitative text analysis [50,51]. The LIWC was first released in the early 1990s and has been updated several times, with the latest version (LIWC-22) released in 2022 [49,50]. This most recent evolution, LIWC-22, is designed to accept written or transcribed verbal text that has been stored as a digital, machine-readable file in one of multiple formats [49]. During operation, the LIWC-22 software processing module accesses each text in the data set and compares the language within each text against the lexicon selected [49].

Owing to its success and practicability, the LIWC has been translated and adapted to its Chinese version (C-LIWC) by humanities and social sciences researchers at the Taiwan Province University of Science and Technology according to Chinese characteristics and culture [52-54]. There are 30 kinds of language-specific words (eg, auxiliary words and prepositions) and 42 kinds of psychological-specific words (eg, positive and negative emotional words), with a total of 6862 words in 72 categories in the C-LIWC [52]. Notably, each word in the C-LIWC has one or more category attributes. Nowadays, there is an increasing number of applications based on the C-LIWC, and it has been used in hundreds of studies across the social sciences, such as psychology, sociology, and communication [28,55,56].

Additionally, nowadays, people manually annotate and build many linguistic knowledge bases. HowNet, a widely used Chinese emotional lexicon, is a typical knowledge base created by the Computer Language Information Center of Chinese Academy of Sciences, which takes concepts represented by Chinese and English words as the description object and reveals the relationship between those concepts and their attributes as the basic content [41,57]. Unlike the C-LIWC, there are no more specific classifications of emotions in HowNet, but it has 17,887 phrases, which are divided into 6 groups based on their emotional tendency: positive evaluation, negative evaluation, positive emotion, negative emotion, perception, and adverb of degree [57]. HowNet has also been widely used in sentiment analysis [53,54].

Various informal text data and the increasing network neologisms on the internet have made it difficult for machines to perform sentiment analysis [15,20]. In addition, there are many polysemous words in Chinese, so the emotional categories of these words need to be judged manually according to the context [44,48,58]. Furthermore, some of the corpora and lexicons are domain specific, which limits their reuse in other domains. Considering that there is no emotional lexicon in the field of BC or even cancer at present, this study aimed to manually construct an emotional lexicon of patients with BC based on the general emotional lexicons the C-LIWC and HowNet and the text corpora of patients with BC in different stages of BC obtained through EW, semistructured interviews, and the Python web crawler of Weibo.

## Methods

### Study Design

**Figure 1:**
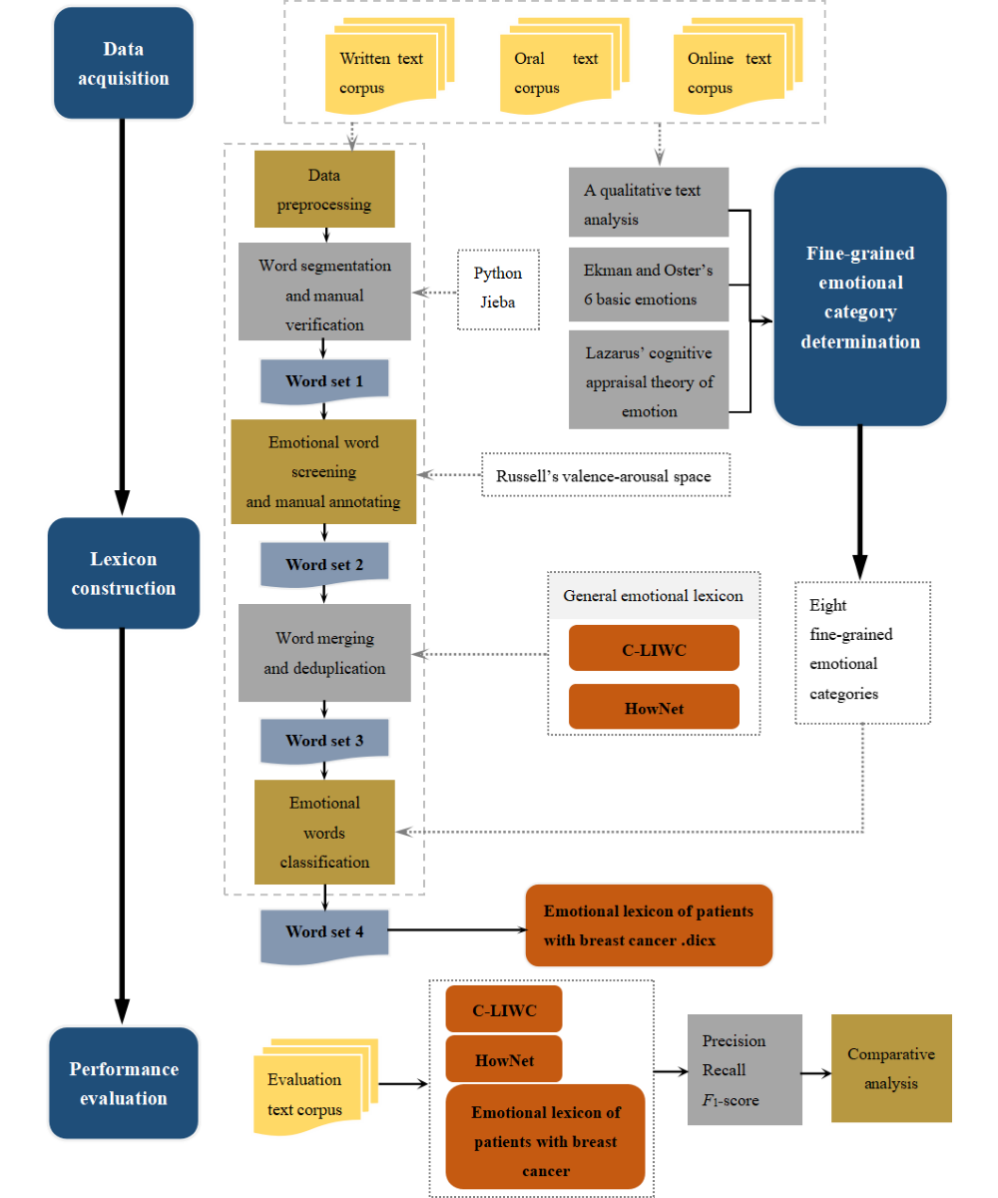
Construction process of the emotional lexicon of patients with BC. BC: breast cancer; C-LIWC: Chinese Linguistic Inquiry and Word Count.

An overview of the construction process of the emotional lexicon of patients with BC is presented in Figure 1. Specifically, to ensure the domain specificity and typicality of words in our emotional lexicon of patients with BC, we used the text corpora of patients with BC obtained from EW, semistructured interviews, and Weibo. Next, drawing upon Goeuriot et al’s [59] approach to domain emotional lexicon construction, we constructed our lexicon by merging the words in the BC text corpora we collected, together with the words in the C-LIWC and HowNet, to ensure comprehensive coverage by the lexicon.

First, all the words obtained from the text corpora after data preprocessing and segmenting were regarded as word set 1 (a Microsoft Excel sheet). Second, based on the valence-arousal space [60], 15 annotators manually judged whether the words in word set 1 were emotional; those words that were emotional and met the inclusion criteria were screened out as word set 2 (an Excel sheet). Third, we combined the words in word set 2 with the words in the C-LIWC and HowNet, removed repeated words, and finally included all the remaining words in word set 3 (an Excel sheet). Fourth, 15 annotators independently labeled the words in word set 3 according to the emotional word categories based on the valence-arousal space [60] and Ekman and Oster’s [61,62] 6 basic emotions. Finally, the labeling results were summarized as word set 4 (an Excel sheet), and then, the words in word set 4 that met the classification criteria of this study were sorted out to form the final emotional lexicon of patients with BC.

In the field of machine learning and data mining, precision (P), recall (R), and the *F*_1_-score are used as performance evaluation indicators of an emotional lexicon [19,20]. Therefore, finally, we compared in LIWC-22 software the results of P, R, and the *F*_1_-score of the positive and negative emotional words identified and classified in BC texts analyzed using the emotional lexicon of patients with BC, HowNet, and the C-LIWC. Results verified the effectiveness of the lexicon construction method used in this paper from the perspective of the identification and classification effect of emotional words.

### Text Corpora Acquisition

To ensure the domain specificity and coverage of the text corpora of patients with BC, EW, semistructured interviews, and the Python web crawler of Weibo were used to obtain the written, verbal, and online corpora of patients with BC. Patients in different stages of BC may experience drastically different emotions and cognitions; therefore, considering patients’ distress peaks and the difference in phase specificity [2,63-65], the text corpora of EW and semistructured interviews of female patients with BC (newly diagnosed, postoperative, or undergoing chemotherapy) were collected. Furthermore, with respect to the potential differences in the phase specificity and acuteness of patient emotions, “newly diagnosed” and “postoperative” were defined as “within 1 month of a new diagnosis” and “1 month postsurgery,” respectively, consistent with previous studies [64,65].

EW participants were recruited from the breast surgery departments of 6 tertiary hospitals in 4 cities of China. Semistructured interview participants were selected from 1 of these 6 hospitals, and they did not participate in EW. Weibo participants were selected from among network users who posted within the supertopic *#breast cancer#*. Since participants on Weibo are anonymous, and we could thus only analyze the texts they disclosed on the internet, we were unable to apply inclusion criteria to this sample. The inclusion criteria for patients with BC included in the EW and semistructured interviews are presented in Multimedia Appendix 1.

#### Acquisition of the Written Text Corpus

Pennebaker’s EW mode [13] was adopted to obtain the written text corpus of patients with BC in 3 different stages: newly diagnosed, postoperative, and undergoing chemotherapy. Consistent with the requirement to include maximal variation during purposive sampling for qualitative text analysis, we recruited 50 EW participants undergoing each of the aforementioned 3 phases [66,67]. Among them, 50 EW texts of participants undergoing chemotherapy were randomly selected from our previous study, a multicenter randomized controlled trial on the effect of prolonged EW on patients with BC undergoing chemotherapy [68]. Other data, including 50 EW texts of newly diagnosed patients and 50 EW texts of postoperative patients, were collected between June 2021 and January 2022. Patients were approached in their hospital rooms by 4 trained female nurse researchers following the guidelines of EW (see details in Multimedia Appendix 2).

#### Acquisition of the Verbal Text Corpus

Objective sampling and snowball sampling were used to select patients with BC. Semistructured, face-to-face interviews were conducted by an experienced female researcher in a quiet conference room in the breast surgery department of a tertiary hospital at the patients’ convenience according to the interview guide (see details in Multimedia Appendix 2). Each interview lasted from 30 to 40 minutes and was digitally audio-recorded. Participant recruitment for the semistructured interviews ended when data saturation was achieved, that is, when no new information emerged [69].

#### Acquisition of the Online Text Corpus

As an emerging multimedia platform, with its advantages (eg, instant, user-friendly to the grassroots, zero-access restriction, high interactivity, weak control, and fission-style mode of dissemination), Weibo has gradually become cybercitizens’ first choice to obtain information and express their opinions in China [15]. Moreover, it also provides platforms for emotion research. Therefore, to collect the online text corpus, we designed a project-developed web crawler in Python language, which used the Weibo application programming interface (API) to systematically scrape the data from June 2021 to February 2022 on Weibo’s supertopic #*breast cancer*#, which involved user nicknames, posts, and comments. We summarized all posts and comments and numbered each text, starting from 1. Referring to the ratio of 7:3 for lexicon construction and the performance evaluation corpus in previous studies [29], 70% of the original online text corpus of Weibo was randomly selected as the construction corpus of the emotional lexicon of patients with BC.

### Text Corpora Preprocessing

Considering the noise in the collected text corpora might affect the accuracy of research, we first integrated and denoised the text corpora obtained from 3 different sources to eliminate the invalid content in the original texts, such as advertisements, blanks, emoticons, punctuation marks, numbers, names of people, and duplicated text.

Next, with the help of the *Jieba* word segmentation toolkit in Python, we segmented the sentence-level corpora into word-level corpora. Due to the particularity of medical words, conventional machine word segmentation may result in the incorrect segmentation of some professional terms. Therefore, we manually verified all the machine-segmented words to reasonably revise any incorrect word segmentation. Given that there is no research on domain lexicon construction in the field of BC at present, we did not have any restrictions on the word frequency length. Thus, we incorporated all the words obtained after segmentation and manual verification into word set 1.

### Determination of Fine-Grained Emotional Categories

At present, the coarse-grained emotional categories of positive and negative are commonly used in most emotional lexicons [15,53]. However, emotions are pervasive among humans, and facial expressions for basic human emotions are identical [61]. For complex and changeable emotional states, more detailed classification is needed to accurately reflect one’s true emotional state. Due to the limitation of emotional categories, coarse-grained categories not only lead to the fuzzy classification of specific emotions but also can only identify a limited number of emotional words, which cannot be effectively applied to the sentiment analysis of emotional information–rich texts in current social network platforms [39]. In contrast, the purpose of fine-grained emotional categories is to analyze more specific and real emotions in individual disclosure texts, such as happiness, anger, and disgust, so as to dig out one’s deeper emotions, attitudes and opinions, and other important information in the texts.

Ekman and Oster’s [61,62] basic emotional categories, Lazarus’ [70,71] cognitive appraisal theory of emotion, and a qualitative text analysis [8] based on all the text corpora we collected were combined to determine the fine-grained emotional categories of the emotional lexicon we constructed. The cognitive appraisal theory of emotion emphasizes that different appraisals and responses to the environment or events will produce different emotions and experiences, which indicates the diversity and complexity of emotions [71]. Ekman and Oster [61] put forward 6 basic emotional states by studying people’s facial expressions (joy, anger, sadness, fear, disgust, and surprise), which have been widely adopted by automatic emotion recognition research institutes in the field of natural language processing [72].

We first summed up 6 basic emotional categories. Furthermore, to improve the accuracy of sentiment analysis in the BC field, we added a seventh emotional category, “somatic symptoms,” based on the qualitative text analysis, the physical symptoms mentioned in the Distress Thermometer (DT) [73], and the MD Anderson Symptom Inventory [74]. Moreover, we found many high-frequency professional medical terminologies of BC through qualitative text analysis, such as “triple negative,” “mastectomy,” and “Her2,” all of which reflect strong emotional and knowledge needs. Therefore, we added “BC terminology” as the eighth emotional category.

To sum up, we finally defined 8 emotional categories, namely joy, anger, sadness, fear, disgust, surprise, somatic symptoms, and BC terminology.

### Emotional Word Screening and Classification Annotation

In this step, 15 annotators, including 11 (73%) medical postgraduates and 4 (27%) nurses, in the breast surgery department with rich clinical experience were invited to manually revise the results of machine segmentation, screen emotional words, and annotate their emotion classification. In addition, we also obtained interannotator reliability scores to determine the accuracy of the annotation. The pivotal point in annotating emotional words is consistency. We used the term “interannotator reliability” to measure the consistency of annotation, which refers to the consistency of different individuals in annotating a particular concept [75]. The Fleiss κ statistic [76] was used to measure the interannotator reliability because it is highly flexible and it can be used for 2 or more categories as well as 2 or more raters [77]. The κ ranges and corresponding consistency strength interpretations were as follows: <0.00, poor; 0.00-0.20, slight; 0.21-0.40, fair; 0.41-0.60, moderate; 0.61-0.80, substantial; and 0.81-1.00, almost perfect agreement [77,78].

#### Emotional Word Screening

All the words obtained after segmentation and manual revising were included in word set 1. First, based on the valence-arousal space [60], 15 annotators were asked to independently manually judge whether the words in word set 1 were emotional. *Valence* represents the degree of pleasant and unpleasant (ie, positive and negative) feelings, while *arousal* represents the degree of excitement and calm. Based on this representation, any emotional state can be represented as a point in the valence-arousal coordinate plane [60]. Annotators marked the words that could arouse their emotional experience or emotional information as “yes,” and vice versa as “no,” and controversial words were marked as “uncertain.” These “uncertain” words were rescreened after discussion by all annotators. After summarizing and integrating the labeling results of all annotators, we stipulated that the words marked “yes” by more than half of the annotators (ie, ≥8, 53%, annotators) would be incorporated in word set 2 based on the research of Wu et al [44] and Zhou and Yang [79]. Referring to the emotional words contained in most existing emotional lexicons [44,72], we found that there are not only some domain-specific emotional words but also most conventional emotional words. In addition, due to the capacity of the constructed lexicon, we combined the emotional words obtained from the corpora with the emotional words in the existing general emotional lexicons to construct an emotional lexicon of patients with BC [59]. Therefore, next, we combined the words in word set 2 with the words in HowNet and the C-LIWC, then removed repeated words, and finally included all the remaining words in word set 3 for the next classification and annotation of emotional words.

#### Classification Annotation of Emotional Words

During this process, 15 annotators manually annotated the emotional category of each emotional word in word set 3 independently according to the 8 emotional categories stipulated in this study. Drawing lessons from the emotional classification standard of emotional words in the C-LIWC and the polysemy of Chinese words (ie, a word may belong to different emotional categories) [44,48], the following provisions were made referring to previous research on manual lexicon construction [44]: (1) the words that could not be classified would be marked as “none”; (2) if the same word was marked by more than 5 annotators in 2 or more categories, this word would be marked as belonging to 2 or more categories based on the research of Wu et al [44] and Zhou and Yang [79]; and (3) if more than 5 annotators marked a word as “none,” and the number of annotators who marked this word in other categories was less than 5, the word would be excluded from the emotional lexicon. Next, we used a random number table to randomly select the annotation results at a rate of 8% for the annotator consistency test [80].

Finally, after the researchers collected, counted, and sorted out the emotional words in each category and the number of people who marked each word in different categories, the emotional words that met the inclusion criteria and the corresponding emotional categories were recorded in word set 4 to form the final emotional lexicon of patients with BC.

### Lexicon Performance Evaluation

In the fields of machine learning and data mining, P, R, and the *F*_1_-score are widely used for classification to evaluate the performance of lexicons [19,20]. The purpose of this step was to compare and analyze the effects of the emotional lexicon of patients with BC constructed in this study and the general emotional lexicons, C-LIWC and HowNet, on text analysis in the field of BC so as to evaluate the performance of the lexicon. The Weibo online text corpus were used for lexicon construction and performance evaluation at a ratio of 7:3 [29]. LIWC-22 software [49] was used to load the emotional lexicon of patients with BC, the C-LIWC, and HowNet and then calculate the 3 variables of text analysis after loading these 3 lexicons. The emotional words in HowNet are only divided into positive and negative emotional categories, while there are many detailed categories in the emotional lexicon of patients with BC and the C-LIWC. Therefore, to maintain the consistency of performance evaluation criteria and comparison indicators, we referred to previous studies [46,48] and stipulated that only P, R, and the *F*_1_-score of positive and negative emotional words analyzed using different lexicons in the same text corpus would be compared. Among the emotional categories in our study, joy is the only positive emotion, while anger, sadness, fear, and disgust all are negative emotions. Considering the polysemy of Chinese words [44,48], we stipulated that all words annotated with “joy” would be divided into the positive category, and any word annotated with one or more categories of “anger,” “sadness,” “fear,” and “disgust” would be regarded as a negative category.

For the convenience of understanding and calculation, we defined the following variables: P, R, and *F*_1_-score. These were calculated using [19]:

- True positives (TPs): number of words judged positive not only by the lexicon but also by manual judgment
- True negatives (TNs): number of words judged negative not only by the lexicon but also by manual annotation
- False positives (FPs): number of words judged positive by the lexicon but judged negative by manual annotation
- False negatives (FNs): number of words judged negative by the lexicon but judged positive by manual annotation

The mathematical formulas of P, R, and the *F*_1_-score are as follows [19,20,47]:


P = TP/(TP + FP)

R = TP/(TP + FN)

*F*_1_ = 2PR/(P + R)

### Ethical Considerations

The research was conducted in accordance with the Declaration of Helsinki and followed ethical principles and guidelines. Ethical approval for the study was granted by the Medical Ethics Committee of Nanfang Hospital of Southern Medical University (NFEC-2021-124), and a standardized informed consent form was established (V1.0/2021-4-14). Eligible participants were informed about the study, and they provided written informed consent for participation prior to the study. During the research, the patients could request to consult an experienced psychologist counselor. Code names were assigned to each participant instead of using their real names.

## Results

### Results of Text Corpora Acquisition

The final emotional lexicon of patients with BC contained a total of 9357 words covering 8 fine-grained emotional categories: joy, anger, sadness, fear, disgust, surprise, physical symptoms, and BC terminology. In total, we collected 150 written texts, 17 interview texts, and 6689 original posts and comments from Weibo, with a total of 1,923,593 Chinese characters.

#### Results of Text Corpora Preprocessing

**Figure 2:**
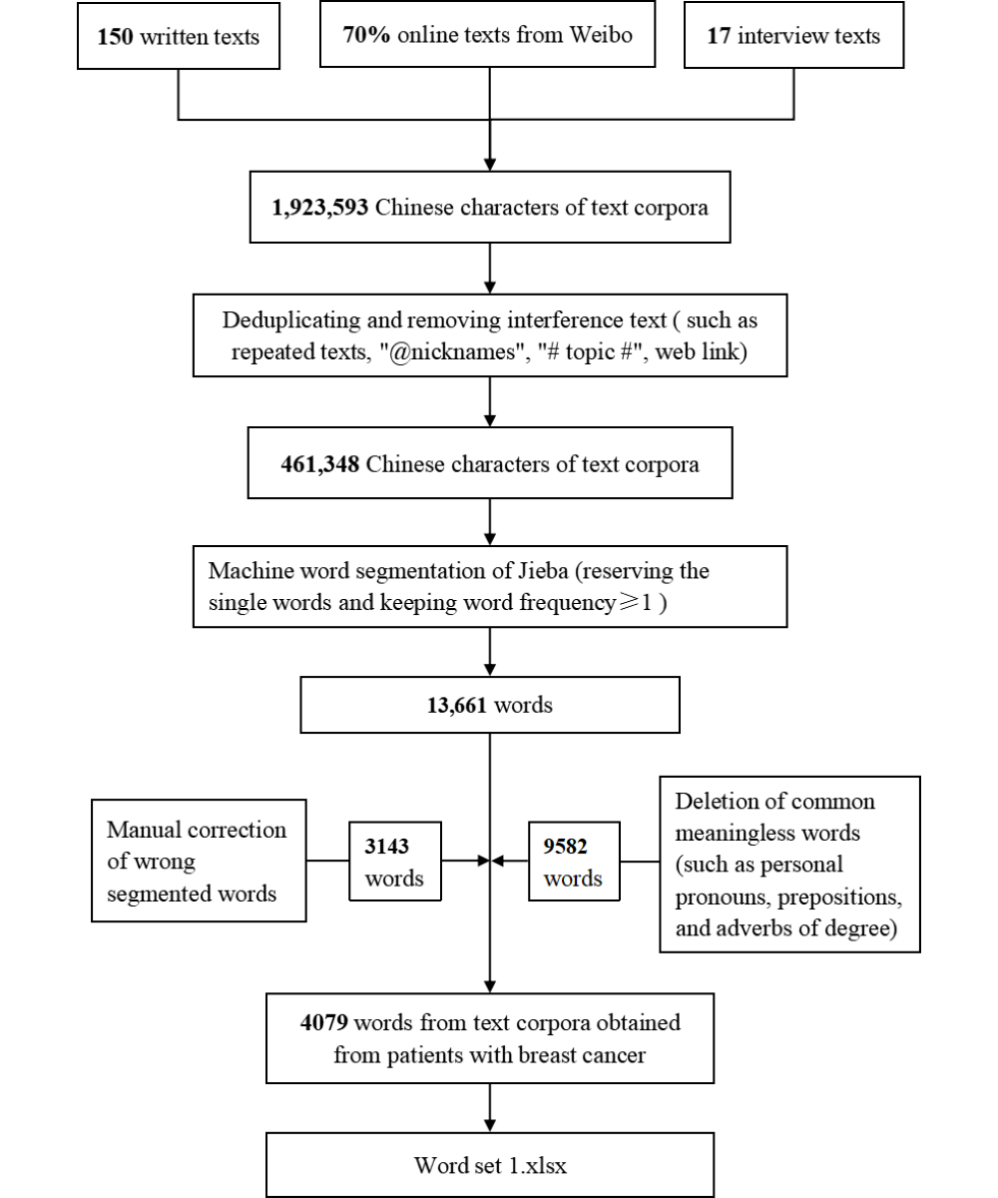
Process of text corpora preprocessing.

First, after deduplicating and removing the website’s automatic comments, spam comments, repeated texts, “@nicknames,” “#topic #,” web links, and other noise data, a total of 461,348 Chinese characters were obtained. Next, all the corpora were subjected to machine segmentation. A total of 13,661 words were segmented (reserving single words and keeping the word frequency≥1). We manually revised 3143 (23.01%) words that were incorrectly segmented by the machine. For example, the word “白蛋白(albumin)” was divided into 2 separate words: “白(white)” and “蛋白(protein).” Afterward, we removed 9582 (70.14%) common meaningless words, such as personal pronouns, prepositions, and adverbs of degree (eg, “you,” “of,” and “very”) after double-checking. Finally, we included 4079 (29.86%) words in word set 1 for the next stage of manual emotional word screening. The detailed flow of text corpora preprocessing is shown in Figure 2.

### Results of Emotional Word Screening and Classification Annotation

#### Results of Emotional Word Screening

In this step, 1829/4079 (44.84%) words were annotated as “no” or “uncertain.” In addition, 2250/4079 (55.16%) words were annotated as “yes”. Among the “uncertain” words, 15 (0.82%) words that met the requirements of our study after a second group discussion were reselected as emotional words. After the first manual annotation by 15 annotators to check whether the words in word set 1 were emotional, we obtained 1998/2250 (88.80%) emotional words that met the requirements of our study. Therefore, a total of 2013/4079 (49.35%) emotional words annotated as “yes” by more than half of the annotators were selected to form word set 2.

We randomly selected the annotation results of 327/4079 (8%) words from the corpora of patients with BC for the annotator consistency test [80], and the Fleiss κ value was 0.491 (95% CI 0.482-0.501), with moderate strength of agreement, which showed that the annotation results were consistent and the consistency was acceptable.

**Figure 3:**
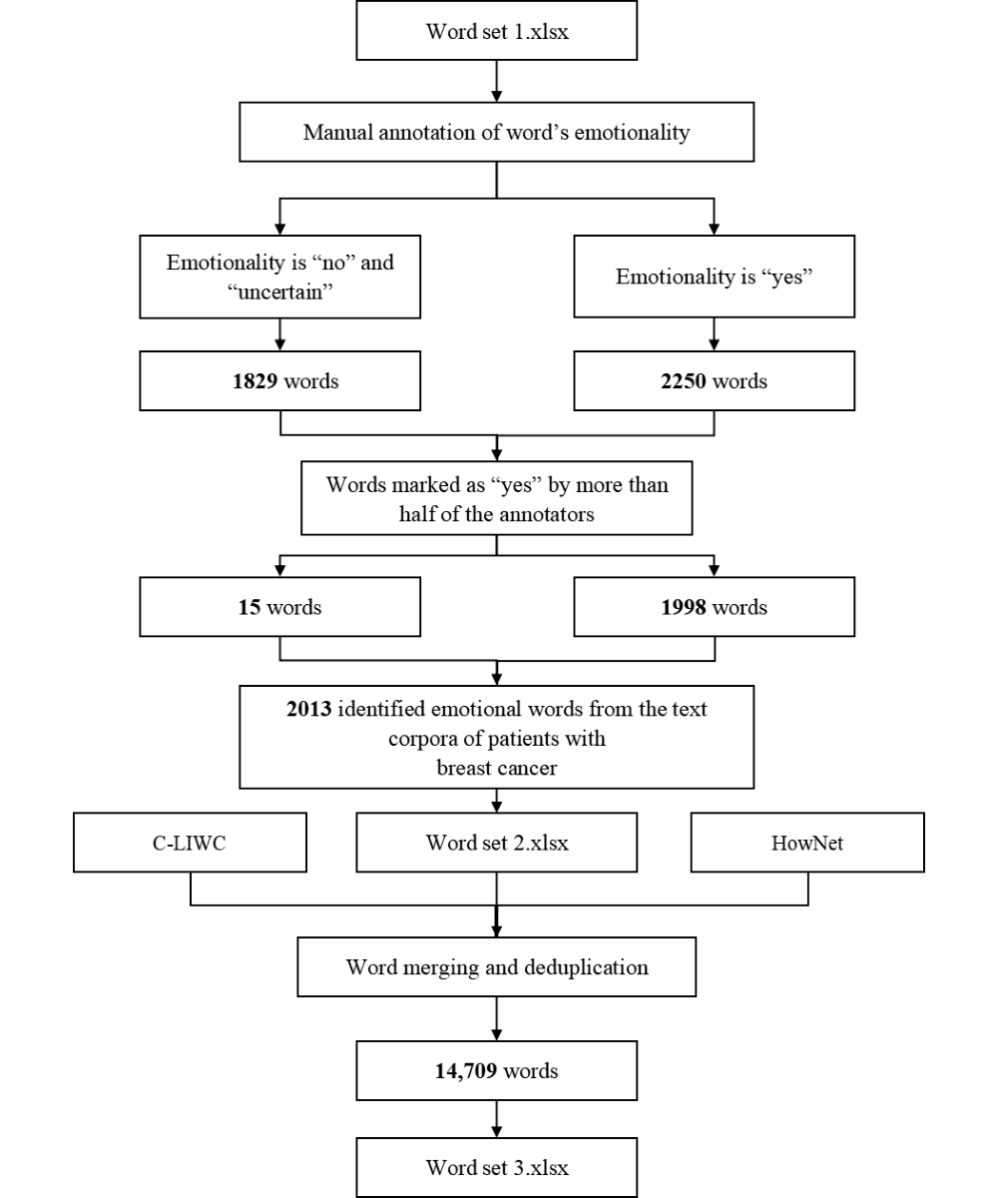
Process of emotional word screening and determination. C-LIWC: Chinese Linguistic Inquiry and Word Count.

In merging the emotional words in word set 2 with the emotional words in the C-LIWC and HowNet, and removing repeated words, 14,709 words were obtained and finally included in word set 3. The detailed process of emotional word screening and determination is shown in Figure 3.

#### Results of Emotional Word Classification Annotation

In this process, 15 annotators marked the emotional words according to the 8 emotional categories specified in this study. The annotating results were collated in word set 4, and those words that met the classification criteria were sorted out to form the final emotional lexicon of patients with BC. The emotional lexicon of patients with BC eventually contained a total of 9357 words reflecting 8 discrete emotional constructs: joy, anger, sadness, fear, disgust, surprise, physical symptoms, and BC terminology. See Multimedia Appendix 3 for the number and examples of emotional words based on 8 emotional categories in the emotional lexicon of patients with BC. We randomly selected the annotation results of 1471 of 14,709 sentiment words at a rate of 8% for the annotator consistency test [80], and the Fleiss κ value was 0.439 (95% CI 0.437-0.441), with moderate strength of agreement, which showed that the annotators’ annotation results were consistent and the consistency was acceptable.

We noted that the 8 emotional categories are not evenly represented in terms of the numbers of items; however, this should not be interpreted as one construct being more prevalent or important than the others but as a natural occurrence of language. Further, we noted that the sum of terms in each emotional category exceeds the total word count of the lexicon; that is because some words/word stems are cross-listed. For instance, “frightened” appears in both the fear and surprise categories; similarly, “berate” is listed in both the anger and disgust categories. Interestingly, some words in the surprise category can reflect both positive and negative emotions, such as “fantastic,” which can be negative when describing a strange phenomenon and positive when describing something wonderful. Additionally, combined with the annotation results and the context in the original BC text corpus, we found that these words in somatic symptoms are often used to reflect morbid problems that have caused obvious disorders in patients with BC, so the emotional arousal degree of these words is high. Furthermore, the words in BC terminology were found to be commonly used objective medical terms in the field of BC, such as physiological and biochemical indicators, chemotherapy plans, and drug names, so the emotional arousal degree of these words is not high. Notably, the emotional valence of words in the somatic symptoms and BC terminology categories could not be generally classified as positive or negative because the words in these 2 categories are a mixture of negative and positive words and some words that have no obvious emotion but are indispensable to sentiment analysis in the field of BC. Thus, the words in the somatic symptoms and BC terminology categories do not belong to any of the aforementioned dimensions, and they are only distinguished by the degree of arousal.

### Results of Lexicon Performance Evaluation

**Figure 4:**
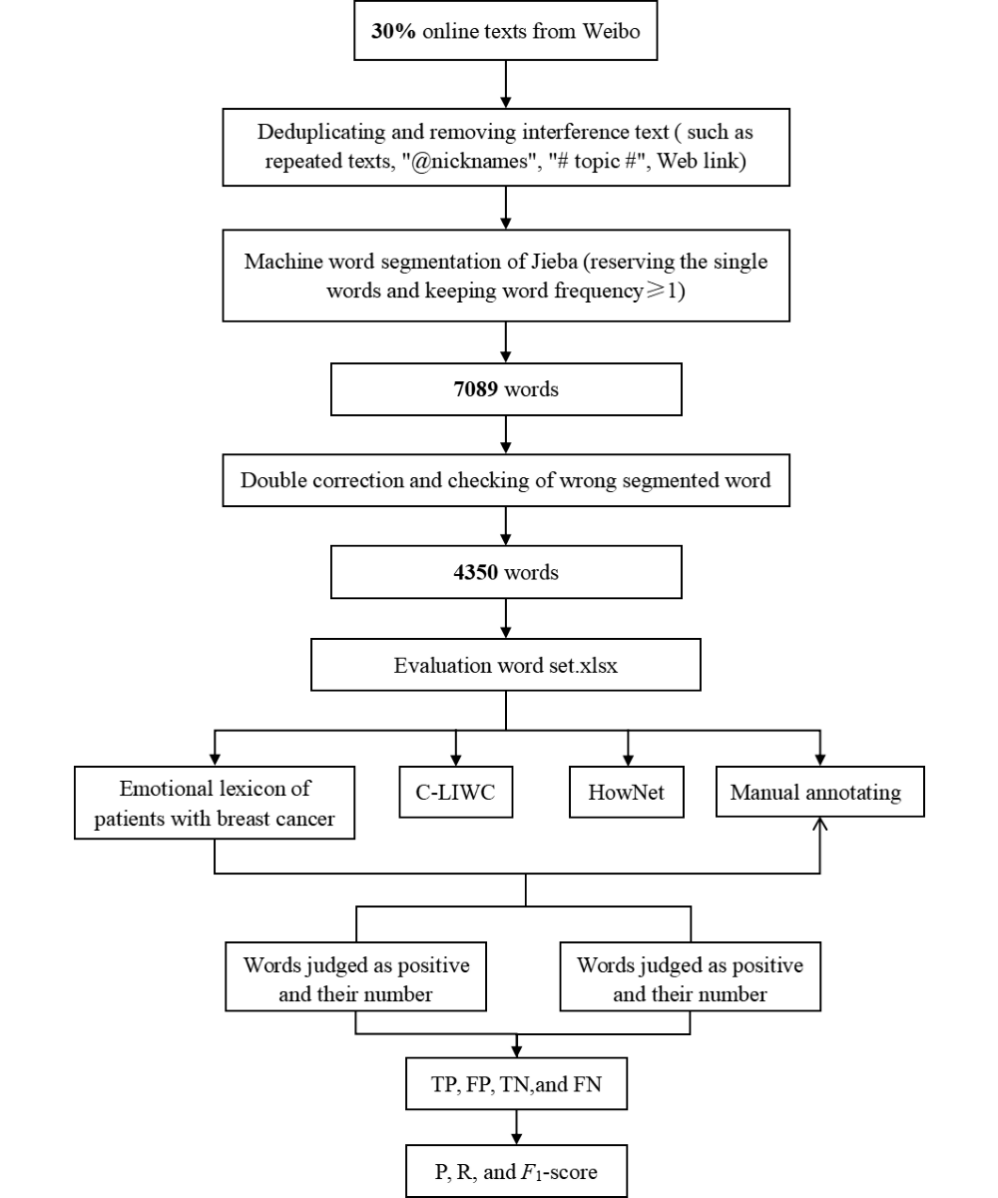
Process of lexicon performance evaluation. C-LIWC: Chinese Linguistic Inquiry and Word Count; FN: false negative; FP: false positive; P: precision; R: recall; TN: true negative; TP: true positive.

Briefly, first, a corpus of 505,868 words from Weibo underwent the same data preprocessing (denoising), and then 7089 (1.40%) words were obtained after word segmentation by the machine. Second, 2 researchers were asked to verify the incorrectly segmented words independently, and then, 4350 (61.36%) words were gathered. Similarly, the 2 researchers annotated the words’ emotional categories specified in our lexicon at the same time, and then a third researcher comprehensively judged the emotional category of each word to obtain manual annotation results. Lastly, the emotional lexicon of patients with BC, the C-LIWC, and HowNet were imported into LIWC-22 software to test the performance of emotional word classification. The detailed process of lexicon performance evaluation is shown in Figure 4. P, R, and the *F*_1_-score obtained after the positive and negative emotional word analysis by the 3 lexicons were calculated and compared. The number of emotional words predicted by the 3 lexicons and manually annotated is shown in Multimedia Appendix 4. The number of positive and negative emotional words predicted by the 3 lexicons that matched the manual annotation is shown in Multimedia Appendix 5. The analysis results of positive emotional words predicted by the 3 lexicons are shown in Multimedia Appendix 6.

As shown in Multimedia Appendix 5, when analyzing the same corpus, 745 words were recognized by our lexicon, which is almost twice as many as the other 2 general sentiment lexicons. In addition, our lexicon had a high recognition rate for both positive and negative emotional words, which is almost consistent with the results of manual annotation. Furthermore, the number of words incorrectly judged by our lexicon and the C-LIWC was less than 10, which indicates that the performance of the 2 lexicons is consistent with manual annotation. However, there were relatively more words incorrectly identified by HowNet.

As shown in Multimedia Appendix 6, P, R, and the *F*_1_-score of the positive and negative emotional word classification by the emotional lexicon of patients with BC were all more than 98%. Specifically, P, R, and the *F*_1_-score of positive emotional words were 98.42%, 99.73%, and 99.07%, respectively, and those of negative emotional words were 99.73%, 98.38%, and 99.05%, respectively. The results of the C-LIWC classification were all over 95% but lower than those of our lexicon. However, it is worth mentioning that the results of these 3 variables for HowNet were slightly insufficient; in the classification of negative emotional words, the 3 values were all less than 95%. In conclusion, our emotional lexicon achieves the best performance in both emotional word detection and classification of the same data set compared to the C-LIWC and HowNet.

## Discussion

### Principal Findings

The overarching goal of this study was to manually construct an emotional lexicon of patients with BC to help researchers identify and analyze terms associated with emotions in a text-based corpus. Consequently, we developed such an emotional lexicon, which consists of 9357 emotional words covering 8 fine-grained emotional categories (joy, anger, sadness, fear, disgust, surprise, somatic symptoms, and BC terminology) related to the emotions of patients with BC. The performance results for P, R, and the *F*_1_-score of the positive and negative emotional word classification by our emotional lexicon were all more than 98%. As expected, the lexicon we constructed outperformed the general sentiment lexicons C-LIWC and HowNet in both emotional word screening and classification of the same data set.

Patients’ ways of emotional expression are diverse [12,16,81]. According to previous related studies [31,82], we also tried to obtain as many corpora of patients with BC as possible through different methods. Finally, 150 written texts, 17 interview texts, and 6689 original posts and comments from Weibo, with a total of 1,923,593 Chinese characters, were collected. These rich corpora are helpful in capturing more special emotional words in the field of BC and ensure the professionalism of lexicon construction. Furthermore, our study also provides a reference for the construction of emotional lexicons in other special fields.

In addition to dealing with some disturbing information in the process of conventional lexicon construction [20,27], we especially added a manual verification step in this process, through which we ensured the correctness and standardization of the words included in the lexicon. Furthermore, unlike the common categories (eg, positive and negative) in most existing general or domain lexicons [20,23,41], in this study, 2 emotional categories with domain specificity, BC terminology and somatic symptoms, were added after analyzing the emotional words extracted from the collected corpora and the emotional categories of existing emotional lexicons. These new special categories are not only a new idea of emotional lexicon construction in the medical field but also a great innovation in emotional category determination.

Moreover, in the process of screening words in patients’ texts, we noticed some differences in the words used by patients in different stages of BC [8,68,81]. Patients in different stages often use terminologies related to the treatment of BC in the current or the next stage and a series of physical symptoms that appear or would appear on their own. The main purpose of this study was to construct an emotional lexicon suitable for all patients with BC and evaluate its performance based on the common text corpora of patients with BC. Therefore, in this paper, we did not conduct a more detailed analysis of the lexicons of patients in different stages of the disease but only introduced and differentiated the main emotional distress of patients in different stages in a qualitative text analysis [8].

### Strengths and Limitations

It is worth noting the strengths of this study. First, to ensure the lexicon’s domain specificity and coverage, EW, semistructured interviews, and the Python web crawler of Weibo were used to obtain written, verbal, and online corpora of patients with BC in 3 different stages of the disease. Second, the emotional lexicon of patients with BC achieved the best performance in both emotional word detection and emotion classification compared to the C-LIWC and HowNet, which proves to be a meaningful input to the early detection and sentiment analysis of patients with BC, providing linguistic insights into identifying patients’ different emotional states.

There are also some limitations. First, the source and quantity of text corpora used for verification were limited. In this study, the construction and performance verification of our lexicon were only based on the Weibo online text corpus, due to which the lexicon’s ability to identify emotional words in Weibo texts is better than that in other online platforms. Second, the data used in the verification stage of this study were segmented words only from patients with BC, not words, sentences, and posts from patients with other types of cancer. Additionally, the emotional intensity values of emotional words in the constructed lexicon could not be determined simply by manual annotation. Thus, the performance of the lexicon we constructed in sentence- and text-level corpus processing and emotion classification needs to be explored and tested in follow-up research combining this lexicon with deep learning to prove the practical value of mental health surveillance of cancer patients. Moreover, although this lexicon significantly outperforms general emotional lexicons, we clearly found that the values of the 3 performance evaluation variables in the emotional lexicon of patients with BC did not reach 100%, which also suggests that the capacity of this lexicon needs to be further expanded and future validation of the lexicon using a large text corpus is necessary.

### Comparison With Prior Work

Interestingly, similar to Gatti et al’s [36] and Wu et al’s [44] studies on lexicon construction, although it is time-consuming to manually construct an emotional lexicon, the precision and coverage of such a lexicon are higher compared to the automatic method. First, in the process of lexicon construction, we manually screened out words that were incorrectly segmented by the machine, thus avoiding the omission of some emotional words [44,48,72], especially words in the somatic symptoms and BC terminology categories.

In addition, as mentioned in many sentiment analysis studies [15,33,35,37,44,58], one of the major challenges in this field is the emergence of a large number of network catchwords. In this study, we found that network catchwords not only appeared in patients’ online texts on Weibo but also were reflected in their written and oral texts to express their emotions, such as “奥利给 (awesome),” “棒棒哒 (good),” and “嘚瑟 (smug).” Therefore, considering the functions of these network catchwords, we naturally included them in our emotional lexicon of patients with BC. However, it is worth mentioning that such words are not included in HowNet, which may be one of the reasons HowNet’s performance on word detection and classification of the Weibo verification text is not high. As for the C-LIWC, there are some commonly used network terms in the lexicon itself, such as “3q (a network neologism whose English pronunciation is similar to “thank you”),” “2傻 (a network neologism where “2” means “stupid and dull” and “2 傻” further emphasizes the person’s stupidity),” and “壕 (nouveau riche)”; thus, it is not difficult to understand that the final analysis results of the C-LIWC are lower than those of our emotional lexicon of patients with BC but higher than those of HowNet.

Furthermore, we suspect that the reasons for the slightly lower performance of HowNet may be the low consistency between the emotional words in this lexicon and the emotional expression in online text. Importantly, there are many emotional idioms and words that are not often used in daily life, such as “杯弓蛇影 (paranoia)” and “首鼠两端 (indecision or vacillation),” which also leads to low recognition of some colloquial expressive words.

Moreover, in line with several previous studies [20,28,35,43], general sentiment lexicons have defects in sentiment analysis of texts in specific fields. Specifically, the proportion of FPs and FNs in HowNet and the C-LIWC is higher than that in our emotional lexicon of patients with BC, which also directly indicates that the polarity of some words is contrary to that in general sentiment lexicons. For instance, the word “任性 (self-willed)” was manually annotated as negative, while it was regarded as positive in HowNet; the word “顽强 (indomitable)” was recognized as negative by the C-LIWC, but it actually expresses positive emotions in the Chinese context. Additionally, comparatively speaking, the experimental results of the C-LIWC are better than those of HowNet, which may be because the classification of words in the C-LIWC is finer and the words are more in line with the psychological and medical fields.

### Conclusion

Instead of constructing a sentiment lexicon automatically, this study aimed to apply a manual construction method to construct a Chinese emotional lexicon in the BC domain based on the commonly used general sentiment lexicons C-LIWC and HowNet. Our emotional lexicon of patients with BC contains both formal emotional words and domain-specific words related to BC. Experiment results showed that the performance of our emotional lexicon is superior to that of the C-LIWC and HowNet in both emotional word detection and classification of the same data set.

We expect the expansion and promotion of this lexicon based on larger corpora and multidimensional methods and the automatic identification, personalization, and accurate management of patients’ emotions based on this lexicon. Meanwhile, more complex construction methods, such as deep neural learning, can be adopted in future research to further improve the proprietary domain lexicon construction method’s portability. We expect that our emotional lexicon of patients with BC will be useful in sentiment detection and will provide more insights into patients’ emotional management and sentiment analysis in terms of emotional need detection among patients with BC.

## Appendix

Multimedia Appendix 1 Inclusion and exclusion criteria for patients included in expressive writing and semistructured interviews.

Multimedia Appendix 2 Pennebaker’s expressive writing instructions and interview guide.

Multimedia Appendix 3 Quantity and representative words of each category in the emotional lexicon of patients with breast cancer.

Multimedia Appendix 4 Number of emotional words predicted by 3 lexicons and manually annotation.

Multimedia Appendix 5 Number of positive and negative emotional words predicted in 3 lexicons that match the manual annotation.

Multimedia Appendix 6 Analysis results of positive and negative emotional words by 3 lexicons.

## Abbreviations

BC: breast cancer
C-LIWC: Chinese Linguistic Inquiry and Word Count
EW: expressive writing
FN: false negative
FP: false positive
GI: General Inquirer
LIWC: Linguistic Inquiry and Word Count
TN: true negative
TP: true positive

## Data Availability

The data sets generated and analyzed during this study are not publicly available due to patient privacy and ethical restrictions but are available from the corresponding author upon reasonable request. The annotated emotional lexicon is publicly available to readers [83], and the password to access this lexicon is available from the corresponding author upon reasonable request.
