# Supplementary material for: Construction of an Emotional Lexicon of Patients With Breast Cancer: Development and Sentiment Analysis
Source: J Med Internet Res. 2023 Sep 12;25:e44897. doi: 10.2196/44897 (PMC10523220; doi:10.2196/44897)
Supplement: Multimedia Appendix 1 [file jmir_v25i1e44897_app1.docx]

**Multimedia Appendix 1.** Inclusion and exclusion criteria for patients included in the expressive writing and semi-structured interview

| **Inclusion criteria** | **Exclusion criteria** |
| --- | --- |
| - female patients diagnosed with breast cancer by pathology | - with any type of psychosis, mental or auditory deficit |
| - age over 18 years | - having other tumors or other major diseases |
| - patients be conscious and can communicate frequently |  |
| - patients who were able to write Chinese and physically able to write by hand for more than 20 min at a time (For expressive writing patients) |  |
| - participants within one month of newly diagnosis, one month after surgery and undergoing chemotherapy |  |
| - voluntary participation and agreed to sign the informed consent form |  |
