# Supplementary material for: Construction of an Emotional Lexicon of Patients With Breast Cancer: Development and Sentiment Analysis
Source: J Med Internet Res. 2023 Sep 12;25:e44897. doi: 10.2196/44897 (PMC10523220; doi:10.2196/44897)
Supplement: Multimedia Appendix 2 [file jmir_v25i1e44897_app2.docx]

**Multimedia Appendix 2.** The Pennebaker’s expressive writing instructions and interview guide

| **Pennebaker’s expressive writing instructions** | **Interview guide** |
| --- | --- |
| Advice your patients to:   - Find a place and time where and when you will not be disturbed. - Write about what you are worrying about or what you have been avoiding thinking about that is affecting your life in an unhealthy way. - Write for a minimum of 20 minutes a day for at least 4 consecutive days. - Write continuously without worrying about proper Chinese or censoring content. - Decide whether to write about the same issue each time or different issues. - Consider reviewing what you are written over time to see how your thinking or emotions have changed. - The information obtained will only be used for this research and will not have any influence on you. | - “Please tell me about your feelings during the stage of your diagnosis. Did you have any thoughts and confusions?” (For newly diagnosed patients) - “Please tell me about your feelings before and after the operation. What adverse symptoms and experiences did the operation bring you?” (For postoperative patients) - “Please tell me about your feelings when you begin to receive or during the chemotherapy. What adverse symptoms and experiences did chemotherapy bring to you?” (For chemotherapy patients) - “What was your feeling during the whole period of illness or treatment?”(For all patients) - “Do you have any new experiences, feelings, or views recently? What things or symptoms have changed your mood?”   (For all patients) |
