# Supplementary material for: Construction of an Emotional Lexicon of Patients With Breast Cancer: Development and Sentiment Analysis
Source: J Med Internet Res. 2023 Sep 12;25:e44897. doi: 10.2196/44897 (PMC10523220; doi:10.2196/44897)
Supplement: Multimedia Appendix 3 [file jmir_v25i1e44897_app3.docx]

**Multimedia Appendix 3.** Quantity and representative words of each category in the emotional lexicon of breast cancer patients.

| **Lexical category** | **Number of words** | **Representative words** |
| --- | --- | --- |
| Joy | 4 081 | cheer (加油), hopeful (有希望的), happy (高兴的), healthy (健康的), be discharged from hospital (出院), recovery (康复), comfortable (舒适的), appreciate (感激), encouraging (鼓舞人心的), propitious (吉利的) |
| Anger | 581 | lose one's temper (发脾气), irascible (暴躁的), quarrel (争吵), nonsense (废话), indignation (愤怒), disobedient (不服从), farting, accusing (指责), fume with anger (七窍生烟), howl (咆哮), berate (怒斥) |
| Sadness | 1 830 | chemotherapy (化疗), depressive (压抑的), hospitalization (住院), give up (放弃), helplessness (绝望), torture (折磨), death (死亡), side effects (副作用), total mastectomy (全切), recurrence (复发) |
| Fear | 1 077 | surgery (手术), anxious (担心的), dreadful (可怕的), metastasis (转移), biopsy (穿刺活检术), frightened (受惊的), nightmare (噩梦), embarrassed (尴尬的), nervous (紧张不安的), hair loss (脱发) |
| Disgust | 2 061 | trouble (麻烦), bored (无聊的), chaotic (混乱的), suspicious (怀疑的), monster (怪物), sarcastic (讽刺的), silly (愚蠢的), puerilely (幼稚地), despise (厌恶), complain (抱怨) |
| Surprise | 165 | surprise (惊喜), stunned (目瞪口呆的), thunderbolt (晴天霹雳), shocked (震惊的), stare blankly (发呆), unexpected (出乎意料的), unprecedented (史无前例的), rare (罕见的), fantastic (荒诞的), abrupt (突然的) |
| Somatic Symptoms | 257 | vomiting (呕吐), dizziness (头晕眼花), nausea (恶心), numbness (麻木), insomnia (失眠), hot flashes (潮热), fatigue (疲乏), constipation (便秘), edema (水肿), ulcer (溃疡) |
| BC Terminology | 235 | tamoxifen (他莫昔芬), docetaxel (多西他赛), zoladex (诺雷得), breast-conserving surgery (保乳术), Herceptin (赫赛汀), TNBC (三阴性乳腺癌), Her2, PICC, BI-RADS4C, TAC |
