# Supplementary material for: Construction of an Emotional Lexicon of Patients With Breast Cancer: Development and Sentiment Analysis
Source: J Med Internet Res. 2023 Sep 12;25:e44897. doi: 10.2196/44897 (PMC10523220; doi:10.2196/44897)
Supplement: Multimedia Appendix 4 [file jmir_v25i1e44897_app4.docx]

**Multimedia Appendix 4** The number of emotional words predicted by three lexicons and manually annotated

|  | Manual annotation | The emotional lexicon of breast cancer patients | C-LIWC | HowNet |
| --- | --- | --- | --- | --- |
| Number of positive words | 587 | 398 | 178 | 357 |
| Number of negative words | 730 | 392 | 208 | 226 |
