# Supplementary material for: Construction of an Emotional Lexicon of Patients With Breast Cancer: Development and Sentiment Analysis
Source: J Med Internet Res. 2023 Sep 12;25:e44897. doi: 10.2196/44897 (PMC10523220; doi:10.2196/44897)
Supplement: Multimedia Appendix 5 [file jmir_v25i1e44897_app5.docx]

**Multimedia Appendix 5** The number of positive and negative emotional words predicted in three lexicons that match the manual annotating

|  | Emotional lexicon of breast cancer patients | | | **C-LIWC^a^** | | | **HowNet** | | |
| --- | --- | --- | --- | --- | --- | --- | --- | --- | --- |
|  | Positive Forecast | Negative Forecast | Total | Positive Forecast | Negative Forecast | Total | Positive Forecast | Negative Forecast | Total |
| Actually Positive | 373(TP^b^) | 1(FN^d^) | 374 | 161(TP) | 7(FN) | 168 | 208(TP) | 10(FN) | 218 |
| Actually Negative | 6(FP^c^) | 365(TN^e^) | 371 | 5(FP) | 177(TN) | 182 | 17(FP) | 164(TN) | 181 |
| Total | 379 | 366 | 745 | 166 | 184 | 350 | 225 | 174 | 399 |

**a**: Chinese linguistic inquiry and word count lexicon; **b**: true positives, **c**: false positives, **d**: false negatives, **e**: true negatives
