# Supplementary material for: Construction of an Emotional Lexicon of Patients With Breast Cancer: Development and Sentiment Analysis
Source: J Med Internet Res. 2023 Sep 12;25:e44897. doi: 10.2196/44897 (PMC10523220; doi:10.2196/44897)
Supplement: Multimedia Appendix 6 [file jmir_v25i1e44897_app6.docx]

**Multimedia Appendix 6** Analysis results of positive and negative emotional words by three lexicons

|  | Emotional lexicon of breast cancer patients | | | **C-LIWC^a^** | | | **HowNet** | | |
| --- | --- | --- | --- | --- | --- | --- | --- | --- | --- |
|  | P^b^ | R^c^ | F_1_ | P | R | F_1_ | P | R | F_1_ |
| Positive | 98.42% | 99.73% | 99.07% | 96.99% | 95.83% | 96.41% | 92.44% | 95.41% | 93.91% |
| Negative | 99.73% | 98.38% | 99.05% | 96.20% | 97.25% | 96.72% | 94.25% | 90.61% | 92.39% |

**a**: Chinese linguistic inquiry and word count lexicon. **b**: precision; **c**: recall; **d**: F-measure.
